# Treating Alcoholism As a Chronic Disease: Approaches to Long-Term Continuing Care

**Published:** 2011

**Authors:** James R. McKay, Susanne Hiller-Sturmhöfel

**Keywords:** Alcohol and other drug disorders (AODD), disease theory of alcohol and other drug use (AODU), chronic disease, treatment, treatment models, treatment outcomes, abstinence, relapse, self-help groups, 12-step-model, continuing care, long-term care, alternative treatment, treatment research

## Abstract

For many patients, alcohol and other drug (AOD) use disorders are chronic, recurring conditions involving multiple cycles of treatment, abstinence, and relapse. To disrupt this cycle, treatment can include continuing care to reduce the risk of relapse. The most commonly used treatment approach is initial intensive inpatient or outpatient care based on 12-step principles, followed by continuing care involving self-help groups, 12-step group counseling, or individual therapy. Although these programs can be effective, many patients drop out of initial treatment or do not complete continuing care. Thus, researchers and clinicians have begun to develop alternative approaches to enhance treatment retention in both initial and continuing care. One focus of these efforts has been the design of extended treatment models. These approaches increasingly blur the distinction between initial and continuing care and aim to prolong treatment participation by providing a continuum of care. Other researchers have focused on developing alternative treatment strategies (e.g., telephone-based interventions) that go beyond traditional settings and adaptive treatment algorithms that may improve outcomes for clients who do not respond well to traditional approaches.

Alcohol and other drug (AOD) use disorders (i.e., AOD abuse and AOD dependence) are substantial public health problems, affecting approximately 10 percent of the population (Grant et al. 2004) and resulting in economic costs to the Nation of around $360 billion annually, with roughly half of this amount attributable to alcohol use disorders (Office of National Drug Control Policy 2004). Add to that the immeasurable costs of suffering for the patients, their families, and those around them, and the enormity of the burden resulting from AOD use disorders becomes even more staggering. The public health effects of AOD use disorders are exacerbated further by the fact that these disorders can be chronic and therefore require constant vigilance by the patients and those around them, as well as repeated intervention. In other words, many patients diagnosed with an AOD use disorder will experience a trajectory characterized by repeated cycles of periods of abstinence alternating with relapse to AOD use that may involve additional treatment episodes. Hser and colleagues (1997, 2007) have used the terms “addiction careers” and “treatment careers” to describe such patterns of recurrent AOD use and repeated treatment experiences.

To account for the chronic nature of AOD use disorders and possibly disrupt the vicious cycle of abstinence, relapse, and treatment, researchers and clinicians are increasingly developing, implementing, and evaluating “continuing care” interventions. These interventions, which may consist of group counseling, cognitive behavioral therapy, or other approaches, are provided for some period of time following the initial acute care episode. Their goal is to stabilize the patients’ situation, lower relapse rates, and thereby also reduce the need for additional treatment episodes. Although this strategy has intuitive appeal, its effectiveness has yet to be determined conclusively. Moreover, several factors interfere with the delivery of continuing care to many patients. These include the following:

- Funding for extended treatment often is inadequate, partly as a result of reductions in treatment duration driven by managed care and other factors.
- Dropout rates are high during the initial phase of treatment, so that only a minority of the patients who begin an acute treatment episode reach the stage at which they could transition to continuing care. In one study, only 50 percent of the patients who began intensive outpatient treatment actually completed the entire 4-week program, and of those who transitioned to continuing care, another 50 percent did not complete that program (McKay et al. 1997*a*).
- Many patients are ambivalent about their need for treatment and only enter a treatment program because of some sort of external pressure (e.g., from family, employers, or the judicial system). These patients may be particularly reluctant to enter a continuing care program.
- Many people are not comfortable or satisfied with the current commonly available treatment options for both initial and continuing treatment, such as group therapy and self-help groups like Alcoholics Anonymous (AA) or Narcotics Anonymous (NA). For example, some patients are not comfortable with the religious focus that traditionally is found in 12-step programs like AA. Others are uncomfortable about sharing their problems or feelings in group settings. And still others may not want to adopt the goal of total abstinence that is a staple of many interventions. These and other factors may lead to early dropout from treatment and thereby also prevent participation in continuing care programs.
- Finally, practical barriers to treatment may prevent or discourage patients from participating in continuing care, such as problems organizing childcare or scheduling work around t reatment appointments as well as lack of appropriate self-help groups in the patient’s vicinity.

For all of these reasons, most patients who begin an acute treatment episode do not receive subsequent continuing care. This makes it difficult for researchers to study the effectiveness of these approaches and, more important, wastes a chance for many patients to break the cycle of abstinence, relapse, and treatment for their AOD use disorders. Consequently, it is evident that new approaches to continuing care are needed. Researchers and clinicians currently are developing new strategies that address some of these barriers. These efforts include more flexible and adaptable protocols, greater attention to the patients’ preferences and needs, use of modern communication technologies, and disease-management approaches that have been proven effective for other chronic medical disorders. This article will introduce some of these newer strategies. After first reviewing traditional approaches to AOD treatment and continuing care and summarizing evidence for their effectiveness, the article explores what is known about how patients can be retained in treatment. It then presents new models of extended treatment and describes some example of new adaptive approaches to long-term AOD treatment and continuing care that have been assessed for their effectiveness. The article concludes with a look at the challenges associated with improving continuing care for patients with AOD dependence.

It should be noted that this review can provide only a selective overview because a full discussion of all available treatment options that might be used as a form of continuing care and of the studies of their effectiveness is beyond the scope of this publication (see McKay [2009*a*] for a more exhaustive review of continuing care research and disease management strategies in the addictions). The studies that were included in the present review were identified through several sources. Literature searches of the Medline and PsychInfo databases were performed using various combinations of the key words “alcoholism,” “alcohol dependence,” “substance dependence,” “continuing care,” “step-down care,” “stepped-care,” “disease management,” and “aftercare.” In addition, the reference lists of identified articles and prior reviews were checked for additional relevant citations.

## Traditional Approaches to Addiction Treatment and Continuing Care

### The Minnesota Model

The traditional treatment of AOD use disorders involves an initial intensive phase in an inpatient facility, followed by a less intense phase that typically is delivered in an outpatient setting, often at a different facility. In most cases, the approach used by these programs is the “Minnesota Model,” a 28-day inpatient/residential rehabilitation program that was developed at the Hazelden Foundation and other residential programs (Anderson et al. 1999; McElrath 1997). It is based on the 12-step AA principles, but with a holistic goal of treating the whole person (i.e., body, mind, and spirit). After completing the program, the patient is referred to AA for continuing care. In addition, the patient may participate in outpatient aftercare group therapy sessions to facilitate the transition from the protected inpatient setting back into the “real world” with all its problems and temptations. Although this approach has been effective for many patients, it suffers from two main drawbacks. First, the approach typically has been relatively inflexible, with little room for adapting to a given patient’s characteristics or needs. In recent years, however, treatment programs based on the Minnesota Model have become more flexible, particularly during the continuing-care phase.

A second limitation of the Minnesota Model is its exclusive focus on the AA/NA principles and philosophy, which are embraced by many AOD abusers but rejected by others. As a result, for patients who are not willing to follow the AA/NA rules and recommendations, the Minnesota Model is not a viable treatment option.

### Outpatient Treatment as Initial Phase

Since the late 1990s, the initial phase of treatment has increasingly been shifted from inpatient settings to day hospitals or intensive outpatient programs (IOPs) (McLellan and Meyers 2004), both to save costs and to make treatment less disruptive to the patient’s life. The basic treatment approach, however, in most cases still follows the Minnesota Model (i.e., is based on a 12-step approach). This phase then is followed by a continuing-care component that frequently is provided at the same facility and uses the same strategies as the initial intensive intervention, just at a lower frequency and intensity.

Currently, most AOD treatment is provided in outpatient settings and only patients with severe coexisting medical or psychiatric problems are treated in inpatient settings. The initial intensive treatment phase typically lasts 30–60 days during which patients attend treatment sessions 2–3 times per week (Substance Abuse and Mental Health Services Administration, Office of Applied Studies 2008). After that, patients enter the continuing-care phase, which typically involves one 12-step– oriented group session per week. In addition, patients are encouraged to attend self-help meetings.

Although initial treatment in an outpatient setting has many advantages, it also has some disadvantages compared with inpatient treatment. For example, a significant percentage of patients participating in IOPs continue to drink or use drugs (e.g., McKay et al. 1997*a*). Patients who fail to achieve at least several consecutive weeks of abstinence during the initial treatment stage have poorer long-term outcomes than patients who do achieve abstinence (Carroll et al. 1994; Higgins et al. 2000; McKay et al. 1999). Therefore, continuing care programs that treat patients who have completed an IOP may have to simultaneously accommodate both patients who have achieved abstinence and those who have not, which may impact treatment effectiveness.

### Types of Continuing Care

Approaches to continuing care that are currently available generally fall into one of three categories: self-help groups, 12-step–oriented group counseling, and individual therapies.

#### Self-Help Groups

Self-help groups such as AA, NA, or Cocaine Anonymous (CA) are the most commonly available type of continuing care for people with AOD use disorders, although they should not be considered formal treatment interventions. All of these groups are based on 12-step programs that provide a spiritual and behavioral guide to self-improvement and offer social support for people seeking to achieve abstinence. Each of these groups offers several types of meetings (e.g., “speaker meetings” with invited speakers, “discussion meetings” in which all participants contribute to the discussion of a given topic, or “12-step meetings” that discuss one of the 12 steps), and participants are encouraged to attend all types of meetings. The composition of regular attendees can vary greatly, and some groups may attract certain subgroups of addicts (e.g., younger people, women, or nonsmokers). Therefore, new members may have to try out several meetings to find a group that is most appropriate for them. In addition, self-help programs with a more secular focus (e.g., SMART Recovery, Rational Recovery, or Save Our Selves [SOS]) are available for those people who are uncomfortable with the religious aspect of AA.

#### 12-Step-Oriented Group Counseling

The most common type of formal continuing care is group counseling based on the 12-step principles. Although the programs are not standardized, they all focus on the 12-step principles underlying the Minnesota Model and self-help groups. During the sessions, participants typically report on their current status (e.g., AOD use) as well as their progress towards working the 12 steps. Other components may include feedback and support from other group members as well as planning of drug-free leisure activities for the upcoming days. The planned duration of this type of continuing care generally is 3 to 6 months; however, dropout rates are high, and most studies have found that about 50 percent of patients stop participating before 3 months (McKay et al. 1999, 2004*a*).

#### Individual Therapies

Although the vast majority of patients receiving continuing care for AOD use disorders participate in group sessions (either self-help groups or formal group therapy), some patients also receive individual therapies, primarily in private-practice settings. Individual therapies rarely are offered in clinical settings, although some therapeutic approaches have been developed for research purposes. These include the following:

- Cognitive-behavioral therapy (CBT) begins with an analysis to identify beliefs, attitudes, and situations that contribute to the patient’s AOD use. Based on this analysis, coping responses that the patient can use are developed and practiced in high-risk situations to avoid relapse (Carroll 1998; Monti et al. 1999). The correction of biased beliefs and attitudes as well as the improvement of coping skills are thought to increase the patient’s self-efficacy, which then may lead to improved coping in high-risk situations and further enhancement of self-efficacy (Bandura 1991). A drawback of the CBT approach is that it requires relatively intensive training for counselors, although a newer, computer-based CBT approach may significantly reduce training times and expenses (Carroll et al. 2008). In one initial study of the computer-based CBT approach, patients receiving this intervention had fewer drug-positive samples during the followup and remained abstinent longer than patients receiving only standard care (Carroll et al. 2008).
- Twelve-step facilitation (TSF) is designed to help patients engage more successfully in 12-step programs (Nowinski et al. 1995). It focuses particularly on the first five steps of the 12 steps,^1^ but also includes other components, such as assessing the patient’s family history of AOD use and the situations that typically lead to AOD use, and providing support for sober living. The TSF program was developed for the Project MATCH study of the National Institute on Alcohol Abuse and Alcoholism (NIAAA), but the extent to which it currently is used is unknown.
- Motivational enhancement therapy is based on the premise that responsibility and capability for change lies within the patient and change must be internally motivated (Miller et al. 1995). Accordingly, the therapist does not guide the patient through the recovery process or offer training in specific skills. Instead, the therapist employs motivational strategies (e.g., feedback on risks associated with current behavior, emphasis on personal responsibility for change, or facilitation of self-efficacy) to increase the patient’s willingness to change his or her behavior (e.g., AOD use).
- Marital and family therapies involve not only the patient but also his or her family. For example, an approach called behavioral couples therapy aims to not only reduce the patient’s AOD use by strengthening the partner’s supportiveness, but also to improve marital satisfaction for both partners (e.g., by increasing shared activities or constructive communication).

### Effectiveness of Current Continuing Care Interventions

Since the late 1980s, 20 controlled studies^2^ have examined the efficacy^3^ of various types of continuing care after completion of inpatient therapy or IOP for initial treatment. Of these, 10 studies included patients with alcohol use disorders and 10 included patients with drug or AOD use disorders. Most of the continuing care approaches evaluated were based on CBT; others involved 12-step group counseling, home visits, interpersonal therapy, and other comprehensive interventions. A systematic evaluation of these studies (McKay 2009*a*,*b*) found that 10 of the studies yielded statistically significant positive results (see table)—that is, one of the treatment groups exhibited a significantly improved outcome on at least one primary outcome measure, with no outcome measure favoring the other treatment group(s).^4^ However, it is important to recognize that a statistically significant difference does not always indicate that the difference is large enough to also be clinically significant.

Despite these caveats, some general conclusions can be drawn from the existing controlled studies of continuing care interventions:

- Studies of more recent interventions were more likely to find positive results than older studies. This suggests that both the interventions and their evaluations have improved in recent years.
- Interventions that had a longer duration (i.e., at least 12 months) or in which greater efforts were made to reach and engage the patients (e.g., through home visits or telephone calls or by involving spouses/partners) appeared to be more effective.

Although the studies provided some useful information, they still suffered from a range of limitations that point to areas to be addressed in future research. First, little is known about the mechanisms that contribute to the interventions’ efficacy in studies with positive outcomes. For example, it is possible that any positive treatment effects observed result primarily from factors that can be found in all interventions, such as an empathic and caring therapist or the structure and support provided by regularly scheduled treatment sessions (Baskin et al. 2003; Wampold 2001). Other investigators, however, have argued that certain interventions derive their efficacy from factors other than those general factors. For example, the positive effects of TSF appear to be mediated by effects on participation in self help groups—in other words, patients receiving TSF are more likely to go to AA meetings, which in turn predicts better outcomes (Longabough and Wirtz 2001). However, more research is needed to identify the factors that account for positive continuing care effects. NIAAA has established a research program on “Mechanisms of Behavioral Change” that is funding work in this important area.

A second limitation is that the rates of participation in continuing care and retention rates throughout the entire program were relatively low, particularly in studies that more closely mirrored real-life conditions. It therefore is important to develop interventions that enhance participation and retention. Some approaches to increasing retention are described in the next section.

Third, the magnitude of the observed effects varied substantially between studies and sometimes was relatively small.

Finally, all of the studies focused on patients who had completed the initial stage of treatment before entering continuing care. However, it is especially those patients who do not complete inpatient therapy or IOP who might benefit most from the lower-intensity continuing-care approaches. Thus, it will be important to design continuing care programs that enroll patients early in the initial treatment process in order to retain them in a continuing care program even if they drop out of initial treatment. Some such programs already exist and will be discussed later in this article.

### How Can Retention in Continuing Care Be Increased?

As indicated above, one of the major problems in the implementation of continuing care is retaining patients for the intended duration of the intervention. Several studies have developed and investigated methods to increase both patient involvement/participation and treatment retention.

A number of correlational and quasi-experimental studies^5^ (e.g., Harris et al. 2006; Hitchcock et al. 1995; Schaefer et al. 2005; Schmitt et al. 2003) have investigated factors that predict involvement and retention in continuing care. These analyses identified a wide range of variables that may have an influence. Taken together, the findings suggest that two general factors may contribute to higher retention rates:

- A combination of certain patient characteristics, including greater problem severity, higher motivation to change, and greater “recovery potential” (i.e., availability of social support; supportive living conditions such as halfway houses; and involvement in pro-recovery activities, such as religious groups); and
- Availability of convenient care situations (e.g., a treatment facility near the patient’s home) and active encouragement from staff during the initial treatment phase (e.g., support from staff in identifying resources and coordinating care).

Several controlled studies also have explored the impact of various strategies to increase initial engagement in continuing care and enhance retention, identifying several procedures and interventions that can have a positive effect. These procedures included the following:

- Case-management strategies, which resulted in longer participation in continuing care (i.e., 43 percent more sessions attended) and improved outcomes in several areas (Siegal et al. 2002).
- Intensive referral to continuing care services that monitored the transition of patients from one level of care to the next. For example, more patients completed intake procedures for the continuing-care programs if they were accompanied by staff members from the initial treatment programs (Chutuape et al. 2001). Similarly, when staff members provided extensive information on available self-help groups and established contact to a group member, patients became more strongly involved in the self-help programs and also had better AOD use outcomes at 6 months (Timko et al. 2006).
- Ongoing encouragement via telephone contacts for up to 12 weeks after discharge from an inpatient program to encourage patients to comply with an agreed-upon continuing-care plan (Hubbard et al. 2007); however, this type of encouragement only generated a relatively small impact.
- Incentives in the form of cash or gift cards, which reliably increased rates of attendance (McKay et al. 2010).
- A multicomponent approach that included a variety of easy-to-implement strategies (i.e., orientation about the continuing-care program, feedback on attendance, reminders to reinforce attendance, behavior contracts, and social reinforcement) resulted in higher rates of treatment completion, longer treatment retention, and higher abstinence rates (Lash et al. 2007).

Taken together, all of these studies indicate that treatment retention can be increased using a variety of low-cost, easy-to-implement measures. Greater treatment retention, in turn, increases the likelihood of positive outcomes. Nevertheless, these traditional approaches do not appeal to or benefit all patients. Therefore, additional continuing care strategies are needed to augment the number of patients with AOD dependence who can participate in continuing care and achieve positive AOD-related outcomes. Some such novel approaches are discussed in the following section.

## Novel Approaches to Continuing Care

Although existing traditional approaches to initial and continuing care for AOD use disorders have been effective for many patients and can be improved further using the strategies outlined above, these approaches still do not engage and/or produce positive outcomes for all patients. Therefore, researchers and clinicians have begun to develop additional programs to increase the number of options available to AOD-abusing patients and their health care providers. This process has focused mainly on extended treatment models that increasingly blur the distinction between intensive initial care and less intensive continuing care aimed at prolonging treatment participation. A second trend is the design of alternative treatment delivery modes that may be able to reach patients with limited access to or interest in traditional settings and strategies. Researchers have begun to assess the efficacy of these new models. However, many of these studies have been conducted in patients with a range of AOD disorders rather than focusing on patients with alcohol use disorders only.

### Extended Behavioral Treatment Models

Several investigators have looked at extending and augmenting currently used behavioral treatment strategies to address specific subgroups of AOD-dependent patients. One group of researchers has focused on the effects of enhanced treatment for homeless people with AOD-use disorders. These investigators conducted a series of studies of a multi-stage therapy including intensive day therapy, followed by reduced-intensity treatment combined with work therapy and access to housing. These benefits were contingent on drug-free urine samples. The investigators found that compared with standard outpatient care, the enhanced treatment resulted in significantly fewer drug-positive urine samples and higher treatment participation (Milby et al. 1996). In a second study, a modified version of this enhanced treatment was compared with intensive day therapy only. Again, participants who were offered abstinence-contingent access to work therapy and housing showed better outcomes (e.g., greater treatment participation, higher abstinence rates, and less homelessness) than participants in the control condition (Milby et al. 2000).

Another study assessed an intensive case management approach that provided a range of services (e.g., help with solving childcare or transportation problems, counseling, outreach activities, and ongoing monitoring) to AOD-abusing women for 15 months. The investigators found that compared with standard outpatient care, the intensive approach resulted in higher levels of treatment initiation, engagement, and retention as well as higher rates of abstinence throughout the study period (Morgenstern et al. 2006). Similarly, an intensive case management approach resulted in better AOD-related outcomes in a different sample compared with usual treatment (Morgenstern et al. 2009).

Thus, extended behavioral interventions have demonstrated some benefits in terms of treatment engagement, participation, and retention as well as with respect to AOD-related outcomes. It is important to note, however, that in many cases these studies compared the extended intervention with some form of “treatment as usual” rather than with a shorter version of the extended intervention. Therefore, it is not entirely clear if the positive effects in these studies are due primarily to the longer duration of the treatment or to the specific components of the extended interventions.

### Extended Telephone-Based Recovery Support

In recent years, some treatment centers have begun to implement telephone-based approaches to supplement and enhance existing continuing care programs. This development was motivated at least in part by findings that although residential treatment centers may develop continuing care plans, many patients will not follow through with these plans once they return to their home communities. To address this problem, centers like the Betty Ford Center in California and the Caron Treatment Centers in Pennsylvania devised telephone-based continuing care programs that involve regular telephone contacts with the patient for up to 12 months.^6^ During these calls, the patient’s AOD use and participation in self-help programs are assessed along with other issues that might contribute to a relapse to AOD use, including psychiatric problems, family problems, exposure to high-risk situations, and participation in health-related activities. This comprehensive review provides both the treatment provider and the patient with an overview of the progress the patient is making towards long-term recovery. An initial analysis of more than 4,000 patients participating in this program at the Betty Ford Center has indicated that greater participation in the program was associated with better outcomes during follow-up (Cacciola et al. 2008).

### Extended Physician Monitoring Programs

One subgroup of AOD-dependent patients that is of particular concern to the public and the medical profession is physicians with AOD use disorders. To maintain their license to practice medicine, these physicians must undergo intensive treatment that is coordinated and strictly monitored by State Physician Health Programs (PHPs) for several years. The patients must maintain abstinence from AODs, are subject to random drug tests to document abstinence, and must adhere to a long-term treatment plan. Any relapses to AOD use or noncompliance with other treatment conditions leads to prompt re-intervention by the PHPs, with the level of the intervention depending on the severity of the relapse/noncompliance (Dupont et al. 2009).

The long-term effectiveness of this intensive and extensive treatment approach was recently evaluated by McLellan and colleagues (2008), who retrospectively examined the records of 904 physicians managed by 16 State PHPs. The analysis indicated very favorable long-term (i.e., 5 years) outcomes for physicians in these programs. Of those physicians with known outcomes, 81 percent completed their contracted period of treatment and supervision. Of those who did complete treatment and resumed practicing, only 19 percent showed evidence of any AOD use over a 5-year followup. Similar results were obtained in a study of physicians in the Washington State PHP who were treated for AOD use problems (Domino et al. 2005). Again, only about 25 percent of the patients had at least one relapse during the follow-up period of up to 10 years, and most of those patients also were able to subsequently achieve abstinence and continue practicing medicine. Thus, both of these studies indicate that continuing care involving extended intensive monitoring can generate positive outcomes, at least in highly motivated patients.

### Extended Self-Monitoring

Another recently developed approach to continuing care relies on self-monitoring—that is, AOD users self-report their AOD use and other factors on a regular basis, which is hypothesized to motivate reductions in AOD use over time. This strategy makes use of such innovative methods as interactive voice response (IVR), whereby participants call into a computer system that prompts them to answer questions via their telephone keypads. Helzer and colleagues (2002) tested this approach in a study of heavy drinkers who were not seeking treatment, asking them to report their alcohol use daily for 2 years. The study found that self-reported alcohol use declined by about 20 percent from year 1 to year 2. Moreover, the vast majority of participants reported at least some decline in their alcohol use, whereas other non-alcohol–related measures did not change. However, this initial study suffered from several methodological limitations, reducing its generalizability. Nevertheless, the findings indicate that this approach warrants further study.

### Extended Medical Monitoring

Because many AOD-dependent patients suffer from a range of (sometimes severe) medical problems related to their AOD use, some investigators have assessed the effectiveness of providing continuing care in medical care facilities rather than specialized addiction treatment facilities. In an uncontrolled study, Lieber and colleagues (2003) evaluated the outcomes of 789 heavy drinkers with severe liver disease, whose treatment was managed in a medical care setting for up to 5 years and included not only comprehensive medical care but also brief interventions for alcohol consumption. The study found that the participants’ alcohol consumption dropped significantly over the study period.

Another study compared the outcomes of alcoholics with severe medical problems who were assigned to standard addiction treatment or to an integrated outpatient care condition that included monthly clinic visits, feedback on the results of tests to track the effects of drinking, counseling using motivational interviewing techniques, family involvement, and outreach to patients who missed appointments (Willenbring and Olson 1999). Patients in the integrated treatment exhibited greater participation in both medical and addiction treatment as well as better alcohol use outcomes. Although further research is needed to investigate this approach, these studies indicate that extended treatment in a medical care setting may be effective for managing patients with coexisting medical problems.

### Extended Pharmacotherapy

Several medications are being used in the treatment of people with AOD dependence. In the treatment of alcohol use disorders, pharmacotherapy relies mainly on two medications^7^:

- Naltrexone, which acts on the endogenous opioid system in the brain, makes the consumption of alcohol less pleasurable in some individuals and also can reduce craving for alcohol.
- Acamprosate, whose exact mechanism of action is not fully understood, appears to reestablish the balance of several brain-signaling systems that are disrupted by alcohol.

Most of these medications are used primarily during the earlier stages of treatment (i.e., for 8–12 weeks). A few studies, however, also have evaluated the effects of extended treatment with naltrexone and acamprosate, with mixed results. One study compared the outcomes of severely alcohol-dependent patients who received placebo or naltrexone for 3 or 12 months (Krystal et al. 2001). After 52 weeks, the study found no significant differences between the three groups in terms of drinking days or number of drinks per drinking days, suggesting that extended naltrexone did not improve outcome. However, a re-analysis of the data from this study did show that naltrexone led to better alcohol use outcomes on another measure (i.e., abstinence versus consistent drinking) (Gueorguieva et al. 2007). Another study assessed the efficacy of two different dosages of an injectable form of naltrexone that only needs to be administered once a month instead of daily and therefore should reduce compliance problems (Garbutt et al. 2005). In this study, patients receiving the higher naltrexone dose showed the greatest reduction in heavy drinking over the 6-month study period. Moreover, the efficacy of naltrexone (e.g., in number of drinking days per month) was greatest in a subgroup of patients who had had at least 4 days of voluntary abstinence before they began treatment (O’Malley et al. 2007). Thus, extended treatment with naltrexone may be most appropriate for certain patient subgroups.

Several European studies that investigated the efficacy of acamprosate using extended (i.e., 12-month) protocols found that the medication can be effective at reducing alcohol consumption in alcoholics following detoxification and that these effects may even persist after treatment with the medication is completed (Carmen et al. 2004; O’Brien and McKay 2006). However, other studies conducted in the United States have not confirmed these findings (COMBINE Research Group 2006). Thus, the efficacy of extended pharmacotherapies in the treatment of alcohol use disorders remains controversial. Clearly, more effective medications and a better understanding of which patients respond best to which medications are sorely needed in order to expand the role of extended pharmacotherapies in the treatment of alcohol use disorders.

### Adaptive Treatment Approaches to Continuing Care

Another relatively recent development in the long-term care of patients with AOD use disorders is the use of adaptive treatment approaches. These approaches are aimed at keeping the patient in treatment for extended periods in a way that minimizes the burden to the patient and treatment staff but allows the parties involved to respond to changes in the patient’s circumstances that alter risk of relapse by changing the intensity of care. Several such strategies have been studied. They fall into three categories: stepped care, extended adaptive monitoring, and adaptive continuation treatments.

#### Stepped Care

In this approach (Breslin et al. 1997, 1999; Sobell and Sobell 2000), patients initially receive the lowest appropriate level of care to minimize the burden on the patient and thus increase treatment participation. If the patient’s response to this level of care is not sufficient, however, or if the risk of relapse increases for some reason (e.g., during a particularly stressful period at work), the frequency and intensity of treatment can be increased. The effectiveness of this approach has been studied in several settings, including treatment of patients with alcohol use disorders in medical settings (Bischof et al. 2008), treatment of patients with opiate dependence (Brooner et al. 2007; Kakko et al. 2007), and treatment of offenders assigned to drug courts (Marlowe et al. 2008). For example, in a German study (Bischof et al. 2008), patients with alcohol use disorders who were treated in medical settings rather than specialized addiction treatment settings were assigned to one of three groups:

- Standard care (i.e., no specialized addiction intervention);
- Full care, which comprised a computerized intervention plus four subsequent telephone-based treatment session; or
- Stepped care, which included the computerized intervention but in which the number of subsequent telephone-based contacts depended on the patient’s response to the initial intervention.

The study found that both the full-care and stepped-care approaches produced better outcomes at 12 months than standard care. Moreover, the outcomes of patients in the stepped-care group were just as good as those in the full-care group, even though overall they only received about half as much treatment as the full-care group. Thus, the stepped-care approach appears to be able to reduce the burden to the patients as well as costs to the health care system without sacrificing treatment effectiveness.

#### Extended Adaptive Monitoring

With this approach, patients initially are monitored at a relatively low frequency, but treatment can be intensified if a patient relapses or appears to be at risk of relapse. One study of such an approach (Foote and Erfurt 1991) found that adaptive monitoring reduced costs and required fewer hospitalizations of AOD-dependent patients compared with standard care.

Scott and Dennis (2002) developed another adaptive protocol referred to as “Recovery Management Checkups” (RMC), in which participating AOD abusers were interviewed every 3 months to assess the need for further treatment. If treatment appeared warranted, as judged by clearly spelled out criteria, the patients were immediately transferred to a linkage manager. This person worked with the patients to help them acknowledge the need for further treatment and address barriers to treatment and who also arranged scheduling and transportation to treatment. Studies found that this approach led to better management of the patients over time and improved AOD use outcomes over the course of the follow-up (Dennis et al. 2003). Additional modifications to address several limitations of the initial studies further enhanced the effectiveness of the intervention (Scott and Dennis 2009).

**Telephone-Based Continuing Care—A Novel Approach to Adaptive Continuing Care**

A relatively novel approach to continuing care of alcohol and other drug (AOD)-dependent patients that is aimed at increasing treatment participation by reducing the burden for patients is telephone-based counseling. Several such interventions have been developed (e.g., Horng and Chueh 2004); this sidebar describes one protocol developed at the University of Pennsylvania (McKay et al. 2004, 2005). This approach ideally should already be initiated while the patient still is in initial intensive treatment, so that the patient becomes familiar with the approach and has the opportunity to build a rapport with the counselor in order to facilitate transition to the less intense continuing care and reduce the risk of dropout from the program. To this end, the patient and counselor should meet face-to-face for one or two sessions, during which the counselor can explain the program, including the structure of the calls and the materials the patient needs to have available during the calls (e.g., self-monitoring worksheets), as well as establish an emergency plan for crisis situations that may occur between scheduled calls. During these orientation sessions, the patient and counselor also should establish a plan to ensure that calls can be conducted as scheduled (e.g., ensure that the patient has access to a telephone and agree on a good time to call and on the steps that will be taken if the patient misses a call).

Once the telephone contacts have been initiated, each contact follows a set protocol that includes the following components:

- Assessment of the patient’s risk and protective factors status at the current time;
- Provision of feedback on the patient’s risk level;
- Review of progress since the last call towards achieving current goals;
- Identification of upcoming high-risk situations;
- Development and practice of coping responses;
- Addressing any problems the patient may currently experience; and
- Setting new goals for the time until the next call.

During these discussions, the counselors can listen for changes in the patient’s behavior (e.g., avoidant, superficial answers) that could indicate that the patient is not truthfully reporting on AOD use and associated problems or is experiencing some problems. By doing this, experienced counselors can get a rather good impression of the patient’s status even in the absence of face-to-face meetings or urine samples.

One important feature of this protocol is its adaptability in response to changes in the patient’s risk status. Thus, if the patient appears at increased risk of relapse, has already suffered a relapse, or does not appear to respond well to the telephone counseling, the frequency of the calls can be stepped up or face-to-face sessions can be scheduled to determine the extent of the problem and ensure that the patient gets back on track toward recovery. Similarly, the protocol allows counselors to modify the content of intervention even without changing the frequency. For example, if during the risk-assessment phase of the call the patient appears to exhibit symptoms of depression, the counselor could implement specific intervention techniques designed to address this.

Finally, it is important to recognize that this telephone-based protocol is not a stand-alone treatment that can be provided instead of clinic-based care. Rather, the protocol is designed to augment and extend treatment following a more intensive intervention. In addition, the protocol is not a substitute for other recommended recovery-oriented activities, such as regular attendance at Alcoholics Anonymous/Narcotics Anonymous or other support groups or other meaningful social contacts away from AOD use (e.g., at church, work, a sports club, or other social or leisure activities). All of these experiences help the patient achieve and maintain abstinence, and changes in the reported relationships between the patient and these support groups can serve as a signal to the counselor that the patient is at increased risk of relapse. Thus, at all times during the telephone contacts, it is important that the counselor be on the lookout for signs of trouble in what the patient says (or does not say), and that the counselor immediately addresses such issues.

—*James R. McKay and Susanne Hiller-Sturmhöfel*

ReferencesHorngFChuehKEffectiveness of telephone follow-up and counseling in aftercare for alcoholismJournal of Nursing Research12111920041513695910.1097/01.jnr.0000387484.40568.bbMcKayJRLynchKGShepardDSThe effectiveness of telephone-based continuing care in the clinical management of alcohol and cocaine use disorders: 12-month outcomesJournal of Consulting and Clinical Psychology7296797920041561284410.1037/0022-006X.72.6.967McKayJRLynchKGShepardDSPettinatiHMThe effectiveness of telephone-based continuing care for alcohol and cocaine dependence: 24-month outcomesArchives of General Psychiatry6219920720051569929710.1001/archpsyc.62.2.199

#### Adaptive Continuation Treatments

Adaptive approaches also can be used in continuation treatments, where the intensity of treatment is reduced for those patients who have shown a good treatment response. Three studies have investigated such approaches to determine which patients might benefit most from different approaches to continuing care. These studies sought to identify aspects of the first phase of treatment—that is, the type of initial therapy or the patients’ response to initial therapy—that could be used to select an optimal continuing care intervention to follow the initial intervention. The results of these studies were as follows:

- O’Malley and colleagues (2003) investigated the outcome of continued naltrexone treatment of alcohol-dependent patients who had received initial therapy consisting of naltrexone plus either primary care-based counseling or specialized alcohol counseling. The investigators found that patients who received primary care-based initial treatment benefited from extended naltrexone, whereas patients who had received naltrexone plus specialized therapy did not benefit from extended naltrexone.
- McKay and colleagues (1997*a*, 1999) compared the outcomes of patients who had completed an IOP therapy and then were randomly assigned either to standard continuing care (i.e., two 12-step-oriented group sessions per week) or to individualized relapse prevention therapy. Overall, there were no significant differences in cocaine- or alcohol-related outcomes between the two groups. Further analyses, however, indicated that patients who were still considered alcohol-dependent at the end of IOP benefitted more from relapse prevention, whereas patients whose alcohol dependence was in remission responded equally well to both therapies.
- In a subsequent study, McKay and colleagues (2004*b*) compared the outcomes of alcohol and/or cocaine-dependent patients who had completed IOP and were randomly assigned to either standard group counseling, individualized relapse prevention, or telephone-based continuing care (for a description of the telephone-based intervention, see the sidebar). The results indicated that the telephone-based approach led to consistently better outcomes (e.g., higher abstinence rates from alcohol and cocaine) than standard care or relapse prevention. Additional analyses (McKay et al. 2005*a*,*b*) found that the degree to which patients had achieved the primary goals of the IOP program (e.g., stopping alcohol and cocaine use, regularly attending self-help meetings, committing to a goal of abstinence, and having confidence in being able to cope without relapsing) was associated with patient response to different types of aftercare. Thus, patients who had failed to achieve most of the goals of IOP did better in the more intense standard continuing care than in the telephone-based intervention. Conversely, patients who had achieved most of the goals of IOP had better outcomes with telephone-based continuing care than with standard care or relapse prevention.
- McKay and colleagues also recently tested an 18-month version of their adaptive, telephone-based continuing care intervention in a sample of 252 alcohol dependent patients who had achieved initial engagement in IOP. Results indicated that compared with patients who received IOP only, those who were randomized to the intervention had significantly better alcohol use outcomes, as indicated by incidence and frequency of any drinking and heavy drinking over the 18 month follow-up. Conversely, a second 18-month telephone intervention that provided monitoring and feedback without any counseling was not superior to IOP only (McKay et al. 2010*b*). Overall, the findings of all the studies discussed in this section indicate that adaptive treatment approaches are at least as effective as other approaches and offer other benefits (e.g., reduced burden on patients and providers and lower cost). These studies also provide information on which patients may benefit most from what type of continuing therapy.

## Conclusions and Future Directions

Researchers, clinicians, patients, and policymakers are increasingly adopting the view that alcoholism and other drug use disorders can be chronic, recurrent conditions and that many affected patients will undergo more than one cycle of treatment, abstinence, and relapse during their drinking careers. As with other chronic medical conditions, long-term care therefore is more and more becoming an integral component of treatment for AOD use disorders. In fact, with the move away from inpatient therapy to outpatient therapy for the initial phase of treatment, the lines between initial care and aftercare (continuing care) are increasingly blurring.

As a result, research to determine the effectiveness of existing continuing care approaches as well as to develop new strategies to enhance patients’ treatment participation and treatment outcome has grown considerably in recent years. These studies already have identified several components of continuing care that contribute to or mediate its effectiveness. These components include longer duration of care (i.e., 12 months or more), active efforts to reach and retain patients in treatment (e.g., by involving significant others, visiting the home, or approaching the patient by telephone), or use of incentives (monetary or otherwise) to retain patients in continuing care for extended periods of time. Moreover, it is important that the treatment focus reaches beyond the patient and his or her AOD use to include the patient’s support systems (e.g., family, friends, employers, or peers), thereby ensuring provision of more integrated services.

One issue that needs to be investigated in this context is how continuing care programs can be designed so that remaining actively involved in treatment becomes a more appealing proposition to patients. The most important goal of treatment obviously is to help the patient live without alcohol or other drugs. This also means, however, that an influence that played a central role in the patient’s life—even if the consequences generally were detrimental— is taken away from him or her, which may lead to a feeling of deprivation. Particularly for patients who do not (yet) suffer the most severe consequences of AOD use and are not ready to change their behavior, such an approach may have little appeal and will not be able to engage the patient’s motivation and participation. Therefore, it is important that treatment participation offers additional benefits to the patient. These could be monetary incentives; support with housing, employment, or AOD-free social activities that are contingent on abstinence; or the feeling of belonging to a supportive community, such as AA. Thus, it is crucial to identify for each patient the most desirable incentives that can motivate him or her to actively engage and remain in therapy. Additionally, patient preferences regarding the type and intensity of treatment (e.g., degree of supervision by others that is acceptable to them) need to be identified to enhance patient engagement and patient satisfaction with both the treatment and the outcomes.

In addition, research should focus on developing treatment algorithms that allow for adaptation of the treatment content and intensity to the patient’s needs and circumstances. Such algorithms would allow treatment providers to determine more accurately which patients would benefit most from which intervention and at which intensity to ensure maximum effectiveness while creating minimal burden for both the patient and the treatment provider. Additional efforts in this context need to be put into designing reliable monitoring tools to keep track of the patient’s progress and signal the need for treatment adaptation.

Another important issue that needs to be addressed, particularly in this age of concern over rising health care costs, is the question of who pays for continuing care interventions. A recent review of studies assessing the cost-effectiveness of continuing care (Popovici et al. 2007) concluded that continuing care models encompassing different treatment modalities can be cost-effective and can yield a cost benefit. However, only a few studies to date have addressed this issue, and all of these had significant limitations. Thus, additional studies looking at the cost-effectiveness and cost benefit of various continuing care models are urgently needed. Further studies need to determine how payment for diverse treatment components can best be coordinated—that is, whether and how funds for continuing care can be shifted between different providers or from other agencies that may have lower expenses if AOD treatment is more effective (e.g., welfare and criminal justice agencies).

The increasing adoption of comprehensive continuing care approaches involving a range of services also necessitates coordination of different components of care, including psychosocial therapy, pharmacotherapy, medical therapy for coexisting medical problems, and adjunct services (e.g., housing and employment support), all of which may be provided by different agencies. As a result, coordination is necessary not only in terms of the logistics of treatment (i.e., who delivers which service at what time and in which setting), but also in terms of how the patient is transferred between different stages of treatment and who ultimately is responsible for the patient’s care. One possible solution is to incorporate continuing-care services into the specialty treatment programs so that the program counselor who works with the patient during the initial treatment phase also is responsible for coordinating the continuing care phase. Alternatively, separate “recovery centers” with their own staff could be established that in one location offer a range of continuing care services.^8^ Finally, continuing care for AOD use disorders could be integrated into medical settings (e.g., primary care clinics) that are already experienced in coordinating the care for patients with other chronic disorders. All of these options have their advantages and disadvantages, and research is needed to determine which approach is most effective and cost-effective.

As this article has shown, much progress has already been achieved in the development of continuing care models that take into consideration the chronic nature of AOD use disorders. If additional issues like the ones outlined above can be addressed by future research, effective disease management approaches are likely to evolve that will allow greater numbers of patients to overcome the debilitating and often chronic condition of AOD dependence.

## Figures and Tables

**Table:** Controlled Studies of Continuing Care Interventions

**Authors**	**Participants**	**Interventions**	**Outcome**
**Studies with positive outcomes**			

**McAuliffe (1990)**	168 opiate addicts in the U.S. and Hong Kong	**Intervention:** Recovery training and self- help group, 3 hours/week for 26 weeks	Intervention group with reduced relapses, lower levels of crime, higher employment rate
		**Control:** Community referrals and/or individual counseling	
		**Follow-up:** 12 months	
**Foote and Erfurt (1992)**	325 predominantly male alcohol and other drug (AOD) users	**Intervention:** Standard continuing care plus 15–20 follow-up contacts	Intervention group with better outcomes on three AOD use–related measures; no differences on three other measures
		**Control:** Standard continuing care	
		**Follow-up:** 12 months	
**Patterson et al. (1997)**	127 male subjects admitted to alcohol treatment for first time	**Intervention:** Nurse visits over 12 months	Intervention group with higher abstinence rates, fewer blackouts, less gambling
		**Control:** Review visits every 6 weeks	
		**Follow-up:** 60 months	
**O’Farrell et al. (1998)**	59 married male subjects treated for alcohol use disorders	**Intervention:** 15 sessions of couples therapy over 12 months	Intervention group with more abstinence days for up to 18 months and better marital outcomes up to 30 months
		**Control:** No continuing care	
		**Follow-up:** 30 months	
**Sannibale et al. (2003)**	77 patients with severe alcohol and/or heroin dependence	**Intervention:** Structured aftercare involving nine sessions over 6 months	Intervention group with better attendance, lower rates of uncontrolled AOD use
		**Control:** Unstructured aftercare, sessions provided as requested	
		**Follow-up:** 12 months	
**Brown et al. (2004)**	194 predominantly male parolees and probationers with opiate and cocaine use	**Intervention:** Aftercare, case management, and crisis intervention for 6 months	Intervention group with higher rates of abstinence from all drugs, less opiate use, lower rates of weekly drug use
		**Control:** No further care	
		**Follow-up:** 6 months	
**Horng and Chueh (2004)**	68 predominantly male Taiwanese subjects with alcohol use disorders	**Intervention:** Five 30- to 60-minute telephone calls over 3 months	Intervention group with higher abstinence rates, better adjustment, lower addiction severity, lower readmission rates
		**Control:** No further treatment	
		**Follow-up:** 3 months	
**McKay et al. (2004b, 2005*b*)**	359 predominantly male patients with cocaine and/or alcohol dependence	**Intervention 1:** 24 sessions standard group therapy	Intervention group 3 with higher abstinence rates than intervention group 1 and higher rates of cocaine-free urine samples than intervention group 2; intervention group 3 with better values on measures of liver function than the other two groups
		**Intervention 2:** 24 sessions cognitive–behavioral therapy/relapse prevention (RP)	
		**Intervention 3:** 12 telephone counseling sessions plus 4 support group sessions	
		**Follow-up:** 24 months	
**Bennett et al. (2005)**	125 predominantly male patients who had completed alcohol treatment but were at high risk of relapse	**Intervention:** 15 sessions of an RP approach plus standard care	Intervention group with lower rates of heavy drinking, fewer drinking days, and a trend toward higher total abstinence
		**Control:** Standard care (3 group sessions per week, social club)	
		**Follow-up:** 12 months	
**Godley et al. (2006)**	183 predominantly male adolescents with marijuana and alcohol use	**Intervention:** 3 months assertive continuing care (home visits, case management, help with employment) plus standard care	Intervention group received more treatment services, had higher marijuana abstinence rates
		**Control:** Standard care (mixed number of sessions)	
		**Follow-up:** 9 months	
**Studies with nagative outcomes**			

**Gilbert (1988)**	96 male alcoholics	**Intervention 1:** Standard 12-month aftercare (weekly or biweekly sessions) with telephone reminders prior to sessions	No group differences on five drinking outcomes. Intervention 2 group had highest attendance rate; better attendance predicted better drug use outcomes
		**Intervention 2:** Standard 12-month aftercare delivered via home visits	
		**Control:** Standard 12-month aftercare without compliance enhancement	
		**Follow-up** : 12 months	
**Ito et al. (1988)**	39 male alcoholics	**Intervention 1:** 8 weeks of weekly group sessions focusing on RP	No group differences on drinking outcomes measures and other variables
		**Intervention 2:** 8 weeks of weekly sessions focusing on interpersonal skills	
		**Follow-up:** 6 months	
**McLatchie and Lomp (1988)**	155 alcoholics	**Intervention 1:** Four mandatory sessions over 3 months	No group differences on relapse rates, Alcoholics Anonymous attendance, other outcomes
		**Intervention 2:** Four voluntary sessions over 3 months	
		**Intervention 3:** Four sessions over 3 months, with start delayed by 12 weeks	
		**Follow-up:** 3 months	
**Hawkins et al. (1989)**	130 primarily male drug abusers	**Intervention:** Skills training and networking activities plus therapeutic community	Only marginally better outcome in interven- tion group on one of six drug use outcome measures; higher skill level at 12 months in the intervention group
		**Control:** Therapeutic community only	
		**Follow-up:** 12 months	
**Cooney et al. (1991)**	96 primarily male alcoholics	**Intervention 1:** 26 weeks of weekly coping skills sessions	No group differences on a variety of outcome measures
		**Intervention 2:** 26 weeks of weekly interactional therapy	
		**Follow-up:** 24 months	
**Connors et al. (1992)**	63 primarily male problem drinkers	**Intervention 1:** Group counseling (eight sessions over 6 months)	No group differences on four drinking outcome measures
		**Intervention 2:** Telephone counseling (eight calls over 6 months)	
		**Control:** No aftercare	
		**Follow-up:** 18 months	
**Graham et al. (1996)**	192 mostly male AOD users	**Intervention 1:** 12 weekly group RP sessions	No group differences on six AOD use measures
		**Intervention 2:** 12 weekly individual RP sessions	
		**Follow-up:** 12 months	
**Project MATCH (1997)**	774 mostly male alcoholics	**Intervention 1:** Four sessions motivational enhancement therapy over 12 weeks	No group differences on two primary drinking outcome variables
		**Intervention 2:** 12 session cognitive–behavioral therapy over 12 weeks	
		**Intervention 3:** 12 session 12-step facilitation over 12 weeks	
		**Follow-up:** 15 months	
**McKay et al. (1999)**	132 cocaine-dependent men	**Intervention 1:** 12-step focused group counseling plus individual RP, two times/week for 20 weeks	No group differences on a variety of outcome measures
		**Intervention 2:** 12-step focused group counseling, two times/week for 20 weeks	
		**Follow-up:** 24 months	

## Footnotes

1 The first five steps focus on accepting oneself as an addict, surrendering to a higher power, and completing a moral inventory.

2 Controlled studies compare the characteristics of two or more groups of patients receiving different continuing care interventions, with the participants assigned randomly or sequentially to the different groups.

3 The term “efficacy” refers to the treatment effects observed in controlled clinical trials, under clearly described conditions. Conversely, the term “effectiveness” refers to treatment effects observed in real-world settings.

4 Conversely, if there were no significant differences between groups for any outcome measure, or if one outcome favored one group and another outcome favored the other groups, the studies were considered to have a negative result.

5 Correlational studies simply examine the relationship between participation in a continuing care program and AOD-related outcomes and therefore cannot be used to determine causality. Quasi-experimental studies compare the characteristics of two or more groups of participants receiving different types of continuing care; however, the participants are not randomly assigned to the different groups but can either choose for themselves which group they prefer or are assigned to groups based on certain characteristics (e.g., severity of drug use).

6 This program, which has been expanded and standardized at Caron, is now known as Recovery Care Services.

7 A third medication, disulfiram also is approved for the treatment of alcoholism. In contrast to naltrexone and acamprosate, disulfiram does not interact with brain signaling systems but inhibits one of the enzymes involved in alcohol metabolism, thereby leading to aversive effects such as flushing, nausea, accelerated heart rate, or shortness of breath. Thus, patients taking disulfiram will avoid alcohol consumption to prevent these aversive effects.

8 Such recovery centers have already been established in the State of Connecticut and the city of Philadelphia.
